# A commentary on'Re: Is ChatGPT a qualified thoracic surgeon assistant?'

**DOI:** 10.1097/JS9.0000000000000880

**Published:** 2023-11-24

**Authors:** Shou-xiang Weng, Hai-hong Zheng, Qing-xin Yu, Jiao-chen Wang

**Affiliations:** a Department of Pathology, Taizhou Hospital, Wenzhou Medical University, Linhai; b Department of Pathology, Ningbo Clinical Pathology Diagnosis Center, Ningbo City, Zhejiang Province, People’s Republic of China

*Dear Editor*,

Large language models (LLMs) have garnered significant attention in recent times, with researchers recognizing their potential to impact various facets of society^1^. Medicine, being an integral part of society, stands to benefit considerably from the capabilities of LLMs. One of the most renowned models in this domain is ChatGPT, which has served as a catalyst for these developments^2–4^. Initially, there were inquiries into whether ChatGPT 3.5 or 4 could replace physicians in clinical settings^1^. However, subsequent studies have focused on assessing ChatGPT’s role as a valuable assistant to medical professionals, recognizing its current limitations. In this context, Pu and colleagues^5^ have highlighted ChatGPT-4’s impressive performance in the field of thoracic diseases, suggesting its potential as a valuable partner for thoracic surgeons. We commend their noteworthy contributions to this discussion. Having reviewed their compelling findings, we would like to offer some suggestions to further enhance this area of research.

LLMs can refine their performance through data input, a process integral to enhancing the model’s capabilities. GPT-4 has the capacity to accumulate knowledge by searching the web. Thus, authors should be mindful that omitting to clear GPT-4’s memory can introduce bias into the results, which should be mentioned^6^. ChatGPT has not undergone training using professional guidelines and real patient therapy procedures, which could substantially enhance GPT-4’s capabilities as a physician assistant. However, ethical constraints prevent the training of GPT-4 with such guidelines and procedures. Consequently, it is advisable to regularly clear GPT-4’s memory history to mitigate potential biases introduced during practice. Furthermore, we observed that Pu and colleagues posed a single question to GPT-4 for each query. This approach may lead to unstable results, as ChatGPT-4 can provide both correct and incorrect answers to the same question^7^. Therefore, we recommend that researchers ask GPT-4 the same question multiple times to obtain a more comprehensive assessment of its performance. Additionally, coordinating the answers obtained can serve as an evaluative criterion for GPT-4’s responses, helping to exclude unstable results that may introduce bias. Implementing these measures can enhance the credibility of results obtained using the current version of ChatGPT. Despite these considerations, GPT-4 holds promise as a prospective assistant for physicians, including thoracic surgeons. It has demonstrated the ability to offer high-quality advice to outpatients, potentially alleviating the workload of surgeons. However, it is imperative that LLMs, such as GPT-4, operate under the supervision and oversight of experienced physicians, taking into account legal and ethical considerations.

Collectively, Pu and their colleagues have furnished valuable evidence on this subject, which greatly advances the prospects of integrating GPT-4 into clinical practice. Our suggestions are intended to enhance the rigor of research in this field. As the utilization of LLMs in clinical practice expands, numerous challenges may arise, necessitating persistent efforts to address them comprehensively.

## Ethical approval

Not applicable.

## Consent

Not applicable.

## Sources of funding

Not applicable.

## Author contribution

J.-c.W. and Q.-x.Y.: designed the writing and revised the manuscript; S.-x.W., H.-h.Z., and Q.-x.Y.: collected and reviewed the literature and wrote the manuscript. All authors read and approved the final manuscript.

## Conflicts of interest disclosure

All the authors declare that there are no relevant conflicts of interest to disclose.

## Research registration unique identifying number (UIN)

Not applicable.

## Guarantor

Qing-xin Yu and Jiao-chen Wang.

## Data availability statement

Not applicable.

## Provenance and peer review

Not commissioned.
